# Homes for Working Boys in London

**Published:** 1893-06-24

**Authors:** 


### HOMES FOR WORKING BOYS IN LONDON.

There are eight of these Homes in London, one and all of them doing good and useful work. The City Home, Pelham House, situated in Spital Square, Bishopsgate, is the oldest and most important of these, and Tuesday, June 13th, was the day fixed for the re-opening, extensive alterations and rebuilding having been absolutely necessary. Amongst others present were the Lord Mayor and Lady Mayoress, the Countess of Darnley, Lady Kathleen Bligh, Hon. Ivo and Mrs. Bligh, Lady Archibald, the Comtesse de la More, Lady Morris, Sir Edward Bradford, Lady Mary Boscawen, the Rev. James Tillard, and the Rev. J. H. Scott.

The Hon. T. H. and Mrs. Pelham gave their guests a hearty welcome, and the Home presented a pleasantly festive appearance. Gymnastic displays were given at intervals by the boys during the afternoon.

Pelham House, which is, as we have said, the Home longest established, was first started some one and twenty years ago with the object of affording a shelter for boys working in the City, who for one reason or another were without respectable homes. Some of the boys come from orphanges or industrial homes, some have drifted from the country, and have no friends in London; many are recommended by some clergyman or missionary. The Homes fulfil a very real want, and a proof of the success and appreciation they meet with is shown by the fact that they are always full, it being, indeed, an almost daily occurrence for admission to be refused on account of want of room. Boys are received from the age when they begin to earn for themselves; the charges are rated according to wages, 4s. 6d. a week is the charge for board, and 2d. in the shilling is added for lodging. The new buildings in Spital Square will accommodate eighty boys. There is a reading-room, library, gymnasium, and dining-room. The dormitories looked most comfortable with their rows of white beds, though we might wish the floor space were somewhat more liberal. For those boys earning better wages tiny cubicles are provided, just big enough to hold bed and chest of drawers.

We noticed a list of "wants" occupying a conspicuous place in one of the rooms, some of which are so very modest that we hope no difficulty will be found in their supply. "Books" head the list, and slippers, left-off clothing, indoor games, knife cleaning machine, &c., are pleaded for. One thousand eight hundred pounds is still needed to complete the buildings and furnishing. We wish the Homes every success, and hope that the help they need and deserve to carry on their labours will be speedily forthcoming.

